# Disruptive Colouration and Perceptual Grouping

**DOI:** 10.1371/journal.pone.0087153

**Published:** 2014-01-22

**Authors:** Irene Espinosa, Innes C. Cuthill

**Affiliations:** School of Biological Sciences, University of Bristol, Bristol, United Kingdom; University of Sussex, United Kingdom

## Abstract

Camouflage is the primary defence of many animals and includes multiple strategies that interfere with figure-ground segmentation and object recognition. While matching background colours and textures is widespread and conceptually straightforward, less well explored are the optical ‘tricks’, collectively called disruptive colouration, that exploit perceptual grouping mechanisms. Adjacent high contrast colours create false edges, but this is not sufficient for an object’s shape to be broken up; some colours must blend with the background. We test the novel hypothesis that this will be particularly effective when the colour patches on the animal appear to belong to, not merely different background colours, but different background objects. We used computer-based experiments where human participants had to find cryptic targets on artificial backgrounds. Creating what appeared to be bi-coloured foreground objects on bi-coloured backgrounds, we generated colour boundaries that had identical local contrast but either lay within or between (illusory) objects. As predicted, error rates for targets matching what appeared to be different background objects were higher than for targets which had otherwise identical local contrast to the background but appeared to belong to single background objects. This provides evidence for disruptive colouration interfering with higher-level feature integration in addition to previously demonstrated low-level effects involving contour detection. In addition, detection was impeded in treatments where targets were on or in close proximity to multiple background colour or tone boundaries. This is consistent with other studies which show a deleterious influence of visual ‘clutter’ or background complexity on search.

## Introduction

The ubiquitous threat of predation has led to the evolution of different camouflage strategies that make an animal difficult to detect or recognize because of its similarity to the background or to irrelevant background objects [1]–[5]. The better the animal matches its background, the less likely it is to be detected by a predator [6].

Although background matching can be highly effective, this alone may not optimize camouflage because, even if an animal matches the background fully, any disparities between the phase of the pattern on the animal and the background, or notably its shadow [7]–[10], might give its location away. As one strategy to overcome the limitations of crypsis, Thayer [11] proposed a theory of disruptive colouration, extended later by Cott [12], which argued that strongly contrasting shapes and patterns can break up an animal’s form, giving the impression of a series of distinct and apparently unrelated objects. A predator might be able to see elements of a disruptively coloured animal, but it might not necessarily identify them as belonging to a potential prey [13]–[15].

**Figure 1:**
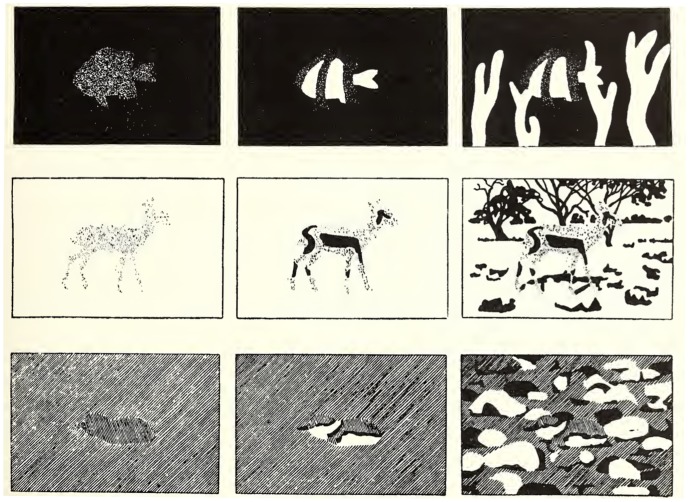
Illustrations from Cott (1940) of the principles of differential blending and disruptive contrast. Note that the form-disrupting effect of high contrast between colour patches within an animal is more effective when these different parts of the animal match different parts of the background (right-hand versus middle pictures). Drawings were reproduced from the book *Adaptive colouration in animals* [12].

**Figure 2:**
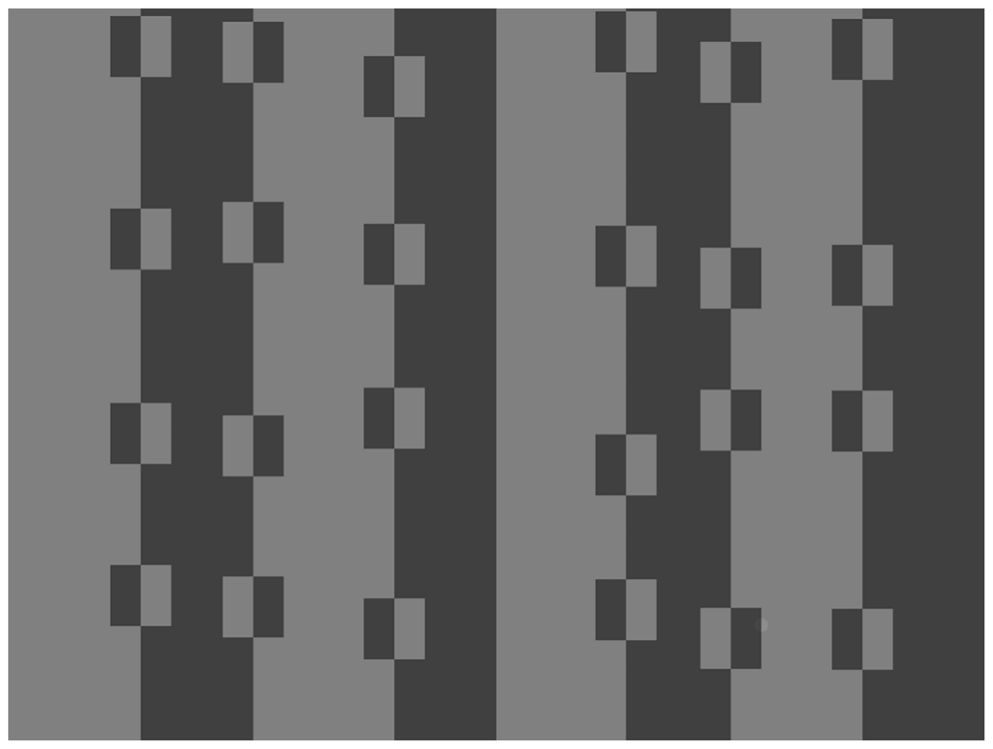
Example of a screen display from the achromatic contrast experiment. The display shows an example from the large square size conditions. The subject has to indicate (by key press) whether the small circular target is on the left or right half of the screen. In this example, the target is located on the right half of the screen (bottom row of squares, second-from-right column) on the right-hand border of a square. We caution that the target itself, being close to threshold detectability when presented on our specific colour-calibrated computer monitor, may not be visible when reproduced at smaller size in the paper and viewed on other displays.

Typical disruptive camouflage, animal or military, places strongly contrasting tones next to each other and, because the outline of an object is a potent cue to both its presence and identity, disruptive patterns at the body’s edge may be particularly effective [12]–[14]. As such, peripheral disruptive patterns have been proposed to exploit edge detectors in low-level vision [15]. The sharp transitions between the adjacent shades in disruptive patterns create false contours within the body that are more conspicuous than the real contours at the body’s edge. For the true outline to comprise a weak edge, significant portions must match the background [15]. Cott [12] described this as ‘differential blending’, proposing that a mixture of background-matching and maximally conspicuous tones might be especially effective (Figure 1). Experiments suggest that Cott was wrong here: mixtures of contrasting, but background-matching, colours are best [16] and very conspicuous unusual colours simply attract attention [17], [18]. Nevertheless, Cott’s general intuition about the importance of differential blending in disruptive coloration seems sound: different patches on an animal should match different patches in the background. As such, disruptive colouration works against perceptual grouping mechanisms [19], [20]. If adjacent colour patches on the animal are more dissimilar to each other than they are to adjacent background colours, then elements of the animal are more likely to be grouped with the background rather than with each other [2]. Given that our brain relies on grouping mechanisms to distinguish an object from the background, we hypothesized that disruptive colouration would be especially effective when different components of the target resemble different objects within the background as opposed to otherwise identical background colours on a single background object. It is noteworthy that in Cott’s original illustrations of the role of differential blending in disruptive coloration, the different colour patches on the animals matched different foreground and background objects (Figure 1). We suggest that differential matching of colours on objects that have already been segmented by the visual system was implicit in Cott’s thinking and this would enhance the effectiveness of disruptive coloration. We tested this proposition by presenting cryptic targets on structured artificial backgrounds to human participants using computer displays (Figure 2). The stimuli were designed so that the target matched the different colours of what appeared to be the same background objects or matched contrasting colours on what appeared to be different objects. In each case however, the level of local background matching, in terms of colour contrast between the target’s edge and the adjacent background, was identical.

In two computer-based experiments human participants had to find a coloured cryptic target hidden on a bi-coloured background. A simple optical illusion, itself reliant on Gestalt principles of contour continuity and feature grouping [19], was used to create scenes (images) in which there appeared to be two-tone striped squares resting on a two-tone striped background. In one experiment two grey tones were used, in the other two hues. The target could be placed on a single background tone, and was monotone itself, or could be placed at the boundary of two tones, and was bi-coloured itself. For (bi-coloured) targets placed at boundaries, the target could be located on either just one single feature of the scene (foreground square or background stripes) or on two (overlapping a foreground square and its background).

We predicted that, first, because of differential blending and disruption, targets on colour boundaries would be harder to detect than targets on homogeneous regions of colour. Second and most importantly, by creating scenes with apparent foreground objects (the squares), targets on boundaries between (illusory) objects would be better concealed than targets on otherwise identical boundaries in colour within a background feature/object (Figure 2). In other words, we predicted that targets located on two objects within the background would be harder to detect than targets placed on just one object within the background, even though the local contrast between the target and the background is identical in both cases. If such an effect existed, this would show that disruptive colouration could be effective through interference with a later stage in visual processing (figure-ground segmentation and object recognition) as well as the low-level mechanism of edge detection.

## Methods

### Ethics Statement

The research had ethical approval from the University of Bristol Faculty of Science Human Research Ethics Committee. All experiments were conducted according to the principles expressed in the Declaration of Helsinki and written consent was obtained from every human participant. The 25 volunteers for each of the experiments were students from the University of Bristol, all participants were naïve, and had normal or corrected-to-normal vision.

We used computer-based experiments where participants had to look at bi-coloured background displays and find a coloured cryptic target. The target was small (16 pixels diameter) and similar in tone to its local background (10% lighter), and so hard to detect. Subjects were told that each display contained one circular target hidden anywhere in the background, then told to press the computer key “A” if the target was found on the left half of the screen, or the computer key “S” if the target was located on the right half of the screen. Displays were created such that target location was unambiguously on the left or right half of the scene. Every subject was presented with four blocks of 50 trials, each block containing a randomised sequence of 5 replicates of each of the 2×5 factorial combinations of treatments (see below). Therefore every subject saw 20 replicates of each treatment combination and 200 screens in total. Subjects were told they could take a break between blocks; in practice none paused for more than a few seconds. All subjects were given 10 practice trials, containing two replicates of each treatment combination (randomly selected) before beginning the experiment proper. Subjects were told to respond accurately and that, if they did not find the target and did not respond within 15 seconds, the computer would advance to the next display. The participants were discouraged from guessing the position of the target but any guesses would have only contributed noise to the data.

There were two experiments; the first was a sequence of grey-scale displays; the second experiment was either a green-red display or a blue-yellow display. There were 25 participants for monochrome experiment 1, and 25 for each of the green-red and blue-yellow displays in experiment 2. In the latter two cases the colours were chosen to be approximately isoluminant (based on CIELab colour space coordinates; [21]). Prior to the experiments, we calibrated the monitors used for the experiment using an Eye-One Pro spectrometer (Xrite Inc., Regensdorf, Switzerland) and calibration software (Colour Management Check-up Kit, Kodak Professional, Eastman Kodak Company 2004). We stress that exact calibration and isoluminance, which if desired would have had to have been determined psychophysically for each test subject, were not necessary components of the experiment.

Displays were 1024 pixels wide by 768 pixels high, with four stripes either side of the midline, which itself was always a colour boundary. Stripe locations were on average at 128 pixel intervals, but exact locations of colour boundaries varied randomly according to a normal distribution of standard deviation 16. This, and the fact that the order of colours (left-to-right A-B-A-B-… or B-A-B-A…) varied randomly with probability 0.5, insured that scenes were variable and targets could not be located by a single change from one trial to the next. Squares were always two-tone with the colour boundary on the midline, and the midline coincident with a stripe boundary on the background. The reversal of colour compared to the background on which each square lay (e.g. square colours A-B on a background stripe B-A) created the illusion of striped squares on a striped background (Figure 2). There were always four squares on each stripe except the midline of the display, with the vertical location of each of the squares varying randomly within each vertical quadrant according to a uniform distribution between 1 and 192 (one quarter of the vertical display size) minus twice the square width. This was done to achieve variable square placing, but with none of the squares too close together.

**Figure 3:**
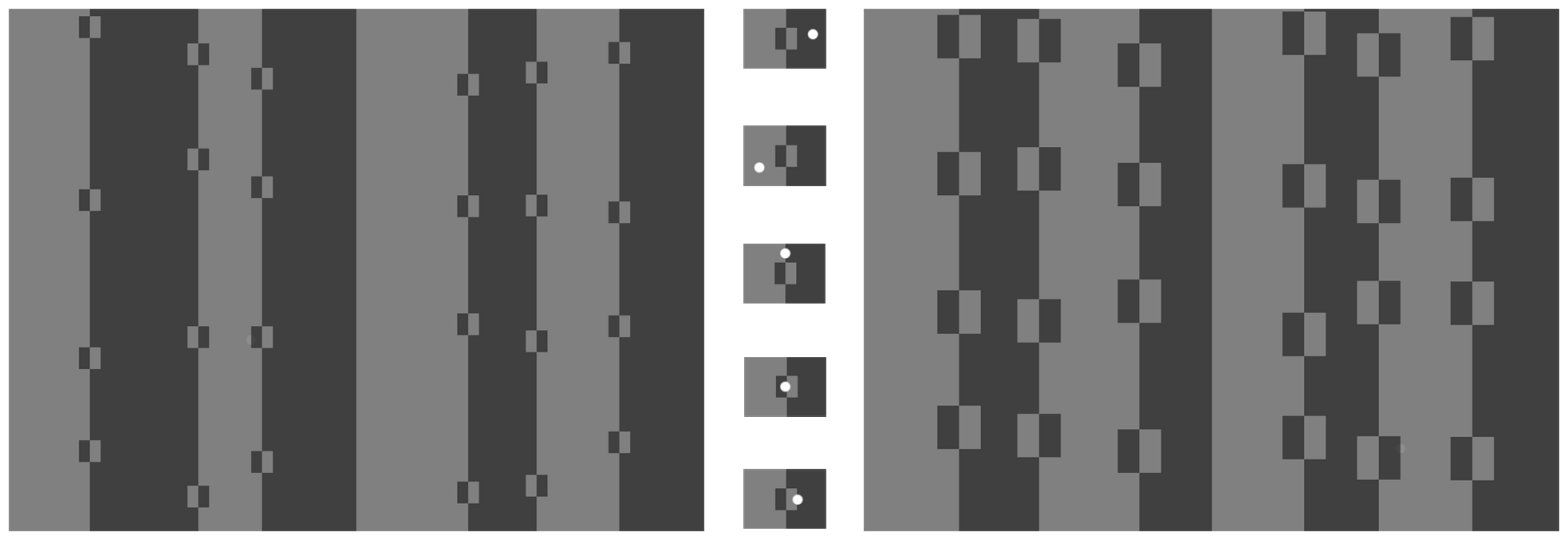
Treatments and examples from the achromatic experiment. The left hand rectangle shows an example from the small square size conditions, while the right hand rectangle shows an example from the large square size conditions. In the middle there are enlarged versions of the five different treatments used, with a white circle used to indicate one of the possible target locations for a treatment (real targets were mono- or two-tone grey, 10% lighter than the immediate background, and placed randomly within the constraints of a treatment); top to bottom: treatments Dark, Light, Stripe, Square, and Border. We used the same square sizes and treatments for the achromatic and the chromatic experiments; the latter involved either (approximately isoluminant) red and green or blue and yellow colour combinations rather than two shades of grey.

Each experiment had a factorial design of two square sizes (small: 32×32 pixels, or large: 64×64 pixels) by five treatments. In the first two treatments, the targets were monotone and on a matching monotone background (dark target on dark background: ‘Dark’; or light target on a light background: ‘Light’). The target was located randomly within a dark or light area comprising any of the background stripes (i.e. on a homogenous dark or light background, and not within a square). In the other three treatments the target fell on the edge of a dark-light boundary and was perfectly in phase with it (dark portion on the dark side of the boundary, light on the light side); however the location of the boundary varied. In ‘Stripe’, the target fell on the border between two stripes (not within or on a square); in ‘Square’, the target lay on the dark-light boundary inside, and the midline of, a square; in ‘Border’, the target lay on the dark-light border between a square and its background (Figure 3). Subject to these placement constraints, the position of a target (which stripe/square, which side, what vertical displacement) was chosen randomly from a uniform distribution.

The screen displays were created with a custom program written in MATLAB (The Mathworks Inc., MA, USA). The software used to display the stimuli and record the responses was Display Master using Direct X (DMDX for Windows; software developed at Monash University, Australia, and at the University of Arizona, USA, by K.I. Forster and J.C. Forster); the software was calibrated to the computer-specific frame and refresh rates using TimeDx by the same authors.

The time taken to detect the target, to the nearest 10 ms, and search success was recorded immediately after the subject pressed the computer key “A” (if target was judged to be on the left half of the screen) or “S” (right half). The mean time to detect the target, and the number of errors (false positives) were calculated for each treatment for each subject. Because of the time-out criterion of 15 seconds, trials where subjects failed to respond within this time period can be considered to be ‘censored’: including these data as ’15 s’ or, even worse, treating them as missing data would lead to an underestimation of true response time. Therefore, although time-outs only occurred in 2.17% of all trials, to get a more accurate estimate of the mean response time for each subject and each treatment, survival analysis was used [22]. Using a custom program to automate the process, and the parametric survival analysis function from Matlab’s Statistics Toolbox, a separate survival analysis was performed for each subject and treatment, fitting a log-normal distribution to the data. The estimated means were then used as the data for subsequent generalized linear mixed models (GLMMs) fitted using the lme4 package [23] implemented in the R environment [24]. Times were modelled with normal error (although raw response time data were skewed, the distributions of estimated means were not); the proportions of trials with errors were modelled with binomial errors and logit link function. The proportions were calculated in relation to trials where a response had been made (i.e. no time-outs), as failure to make a response is a different (and rare) class of error compared to a wrong decision. Initial GLMMs included square size (two levels), treatment (five levels), plus their interactions, as fixed effects and subject (random intercepts) as a random effect. Significance of terms was tested by the change in deviance between models with and without the term in question, using a chi-square distribution [23]. Pair-wise contrasts between levels within a significant factor were tested with t-tests. The key *a priori* treatment comparisons are with Border, so we tested all four other treatments against this using a matrix of simple contrasts (tested simultaneously and without correction for multiple testing). Additional pair-wise comparisons (e.g. of two-tone treatments with monotone, or square vs stripe) are of secondary interest and so were corrected for simultaneous multiple comparison using the Tukey-type procedure in the R package multcomp [25].

We note that analysis of the data using classical univariate ANOVA on mean response time and arc-sine-square-root transformed proportions (subject as a random effect, all other effects fixed), with no model simplification, yields very similar conclusions in terms of the magnitude of effects and which are statistically significant. We present the GLMM results because, in particular for the analysis of errors, they yield more precise estimates of the effects of interests (due to their higher power).

## Results

### Achromatic Experiment

**Figure 4:**
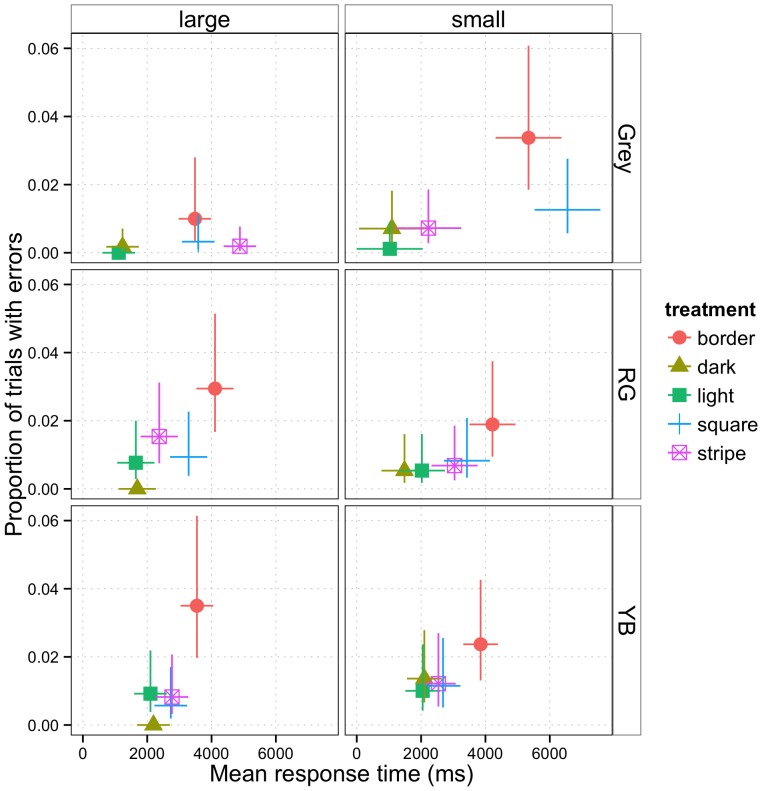
Mean error rates and response times as a function of treatment, square size and experiment. In separate experiments, backgrounds and targets consisted either of two greyscale tones (‘Grey’), approximately isoluminant red and green (‘RG’) or yellow and blue (‘YB’) shades, with squares either small or large in size (see Figure 2 & Figure 3). Estimated means and standard errors for each treatment were taken from generalized linear models. Where standard errors are not visible it is because they are smaller than the symbol representing the mean.

We found a significant interaction between square size and treatment on the time taken to detect the target (*X^2^* = 63.49, *df* = 4, *p*<0.0001). For the small square size displays, treatment was significant (*X^2^* = 80.97, *df* = 4, *p*<0.0001) and subjects took longer to find targets located on boundaries than on monotone backgrounds (Figure 4; Table S1). The difference in time taken to detect the target between treatments can be summarised as (Square = Border)>Stripe>(Dark = Light) (Table S1). For the large square size displays treatment was also significant (*X^2^* = 118.71, *df* = 4, *p*<0.0001) and the differences in time can be summarised as Stripe>(Square = Border)>(Dark = Light) (Table S1); it is the reversal of the relative times to find the target in the Square and Stripe treatments that accounted for the treatment*square size interaction in the first analysis. There was no significant interaction between square size and treatment in the proportion of errors made (*X^2^* = 1.06, *df* = 4, *p* = 0.901), nor was there a main effect of square size (*X^2^* = 2.64, *df* = 1, *p* = 0.105). However, errors did vary depending on the treatment (*X^2^* = 69.11, *df* = 4, *p*<0.0001), with the highest error rate being for targets located on borders; (Figure 4,Table S1; *p*<0.0001 for border vs. other treatments). In other *post hoc* comparisons, Square had more errors than Light but all other comparisons were non-significant (Table S1).

### Chromatic Experiment

In the Red-Green displays, there was no significant interaction between treatment and square size for the time taken to detect the target (*X^2^* = 2.97, *df* = 4, *p* = 0.563), nor was there a main effect of square size (*X^2^* = 1.63, *df* = 1, *p* = 0.202). Treatment was significant (*X^2^* = 99.52, *df* = 4, *p*<0.001), with the times taken having the pattern Border>(Square = Stripe)>(Dark = Light) (Figure 4; Table S1). There was no significant interaction between treatment and square size for the proportion of errors made (*X^2^* = 7.47, *df* = 4, *p* = 0.113), nor a main effect of square size (*X^2^* = 0.64, *df* = 1, p = 0.422). Treatment was significant (*X^2^* = 30.55, *df* = 4, p<0.0001) and the difference in errors between treatments can be summarised as Border>(Square = Stripe)>(Dark = Light) (Figure 4; Table S1).

Looking at the Yellow-Blue displays, we did not find a significant interaction between square size and treatment for the time taken to detect the target (*X^2^* = 2.22, *df* = 4, *p* = 0.695), nor a main effect of square size (*X^2^* = 0.05, *df* = 1, *p* = 0.829). However, there was a significant effect of treatment (*X^2^* = 78.91, *df* = 4, *p*<0.0001), with decreasing detection times in the order Border>(Square = Stripe)> = (Dark = Light) (Figure 4; Table S1; the> = notation here indicates that Stripe was significantly different from Light at *p* = 0.025 but not significantly different from Dark at *p* = 0.068). There was a significant interaction between square size and treatment on the proportion of errors made (*X^2^* = 15.155, *df* = 4, *p* = 0.004). Differences in the proportion of errors were not significant between treatments with a small square size (*X^2^* = 4.488, *df* = 4, *p* = 0.344), but were for large (*X^2^* = 41.15, *df* = 4, *p*<0.0001). The difference in errors between treatments can be summarised as Border>(Square = Stripe = Light)> = Dark (Figure 4; Table S1), although the comparisons with treatment Dark could not be computed due to zero errors, and so no variance, in this treatment.

## Discussion

Two main patterns are evident across the achromatic and the two chromatic experiments. First, the quickest targets to locate, with lowest error, were monochromatic targets placed on a single matching background colour. This could be because matching multiple background objects is better than matching one, even when the local contrasts at the target edge are identical, and is what we might expect from disruptive coloration. Dimitrova and Merilaita [26] had similar findings with blue tits searching for artificial targets; targets which bore multiple elements found in the background were harder to find than those bearing fewer background elements. However, in our experiment, as the hardest-to-detect targets (Stripe, Square, Border) were also all on colour boundaries, this could also be consistent with a crowding effect, which we discuss later.

Our second result and the motivation behind the experimental design, is that as predicted, subjects often found it more difficult to detect targets on the border between the (illusory) foreground squares and the background than targets on boundaries with identical local contrast that lay within squares or on background stripes; subjects made more errors in all experiments. Moreover, in the two chromatic experiments they took longer to find the targets, whereas in the achromatic experiment they took a similar amount of time for treatments Border and Square. Our interpretation is that targets with colours matching two (apparently) different objects were harder to detect than targets matching two colours on the same (apparent) object. This provides evidence for differential blending, whereby a benefit exists for having patches with a mixture of contrasting colours or luminance that match different patches in the immediate adjacent surroundings. More importantly, the benefit is greater if those adjacent background colours are perceived as belonging to objects that are themselves distinct. As such, we argue that disruptive colouration exploits perceptual grouping mechanisms; if neighbouring colour patches on the target are less similar to each other than to those on neighbouring background objects, then the target’s patches are more likely to be grouped with the different background objects rather than with each other, making segmentation of the target very difficult. While the camouflage literature has frequently referred to interference with higher cognitive functions, including attention, the only mechanism that has been experimentally investigated is interference with edge-detectors [15]. In showing that highly contrasting patterns when combined with differential blending are effective in exploiting perceptual grouping mechanisms, our results indicate effects beyond the lowest level in visual processing.

In both the achromatic and in the chromatic experiments, targets located near or within other objects were harder to detect, particularly when in a constricted space (within the small squares or on background stripes between the large squares). This is consistent with a crowding effect, known to have a negative influence on object recognition tasks in humans (for the predicted role in camouflage see [19], [27], [28]). Crowding is generally defined as “the deleterious influence of nearby contours on visual discrimination” [29]; in other words, crowding effects impair the ability to recognize objects in clutter [29]–[31]. Crowding effects are typically observed in peripheral (non-foveal) vision when there are features in the background in close proximity to the target, and these background features are similar in type to features on the target (such background features are termed distractors). Therefore it is reasonable to think that crowding effects could explain some of the treatment differences observed in our experiments. Interestingly, in the achromatic experiment, the relative response times for the Stripe and Square treatments were reversed in the large and small square treatments (Figure 4). Response times were longest for treatment Square when squares were small (and so the proximity of colour boundaries, near the midline of the squares on which targets were placed, was greater); conversely response times were longest for Stripe when squares were large (and so the length of available stripes, between the squares, on which targets could lie was smaller; the corollary being that colour boundaries on squares were again closer). This reversal is consistent with a crowding effect. Crowding could not, however, explain the greater difficulty in locating targets on square-background boundaries (treatment Boundary) compared to targets on boundaries within a square (treatment Square) seen in both error rates and response times in the two chromatic experiments, and error rates (though not response times) for the achromatic experiment. In the Square treatments, there were more nearby and surrounding contours and so the crowding effect should have been larger than for the Border treatment. The congruence of disruptive contours in the target with perceptual segregation of square from background therefore seems to have had an independent effect on top of the effects of visual clutter/complexity in our study.

In conclusion**,** a potent form of camouflage is to combine highly contrasting markings near the edge of the body with differential blending. Disruptive colouration works by breaking the form of the animal through the use of high contrast colours and/or luminance. Differential blending groups different patches of the animal’s body with different shades/colours in the background. We perceive individual objects as possessing multiple attributes or features. A critical task of the visual system is to bind those features into a single percept; however, feature binding can fail, resulting in the experience of illusory conjunctions of physically disjointed features. With this study we provide results suggesting that is even better to group different patches of the target’s body with different objects in the background and not rely solely on matching those patches to various background shades.

Given his drawings (Figure 1), it would not surprise us if Cott (1940) had matching multiple background objects in mind when defining components of disruptive coloration such as differential blending. Classic military disruptive patterns, such as US Woodland or British DPM, also employ sharply contrasting green and brown tones; we would suggest it is relevant that these are not simply two colours found in the background, but the colours of distinct background objects: vegetation and earth. We note also that an additional interpretation of our findings is that the hardest-to-find objects (treatment Border) blend differentially with background features that are perceived as lying in different depth planes (the squares are perceived as lying on top of the striped background). Cott’s drawings also illustrate animals matching background objects in different depth planes and, in a separate section of his book, argued strongly that animals use shading to create depth illusions. Co-location in the same depth plane contributes to perceptual grouping [32], [33], so using colour to disrupt this percept should be advantageous [20]. Some previous experiments on disruptive coloration, where two-tone 2D moth-like targets were placed on the 3D textured background of tree bark [13], [16], [18], could also be interpreted in this way: the light colours of the target match the raised bark and the dark colours match the shadows of the furrows between the ridges. The possible distinct benefits of matching objects in different depth planes deserves further investigation; neither our experiment, nor those above that we suggest could be interpreted this way, were designed to isolate any such effect. Distinct from the effects of differential blending, our results also suggest animals would be better concealed if they hid near strongly contrasting edges and other prominent features in the background. This echoes other studies showing reduced detection of prey on complex backgrounds [26]–[28] and can be usefully linked to the literature on visual clutter [30].

Our findings suggest that disruptive colouration might interfere with later stages in visual processing in addition to previously demonstrated effects involving low-level contour detection [15]. We would suggest that disruptive coloration can be usefully defined by interference with perceptual grouping mechanisms [19], [20]. This is implicit in most accounts of disruptive coloration, and we feel that this is what most people mean by colours that “break up shape and form”. This approach also helps in distinguishing this form of camouflage from other mechanisms such as background matching and distraction patterns.

## Supporting Information

Table S1 **Pairwise comparisons between treatments in the time taken to detect the target and the proportion of trials with errors.**
(DOCX)
