# Supplementary material for: Disruptive Colouration and Perceptual Grouping
Source: PLoS One. 2014 Jan 22;9(1):e87153. doi: 10.1371/journal.pone.0087153 (PMC3899390; doi:10.1371/journal.pone.0087153)
Supplement: Table S1 — Pairwise comparisons between treatments in the time taken to detect the target and the proportion of trials with errors. (DOCX) [file pone.0087153.s001.docx]

**Table S1.** **Pairwise comparisons between treatments in the time taken to detect the target and the proportion of trials with errors.**

(a) Time (values are t statistics with df = 96 and associated p-values).

| Experiment: | Greyscale | | Red-Green | Yellow-Blue |
| --- | --- | --- | --- | --- |
| Square size: | Small | Large | Both | Both |
| **Border - Dark**^1^ | **6.45, p<0.001** | **7.18, p<0.001** | **9.46, p<0.001** | **8.14, p<0.001** |
| **Border - Light**^1^ | **6.56, p<0.001** | **7.55, p<0.001** | **8.55, p<0.001** | **8.52, p<0.001** |
| **Border - Square**^1^ | **1.84, p=0.069** | **0.32, p=0.747** | **2.96, p=0.004** | **5.20, p<0.001** |
| **Border - Stripe**^1^ | **4.73, p<0.001** | **4.45, p<0.001** | **5.35 p<0.001** | **5.48, p<0.001** |
| Dark - Light | 0.10, p=1.000 | 0.38, p=0.996 | 0.91, p=0.893 | 0.39, p=0.995 |
| Dark - Square | 8.30, p<0.001 | 7.50, p<0.001 | 6.50, p<0.001 | 2.94, p=0.033 |
| Dark - Stripe | 1.72, p=0.425 | 11.63, p<0.001 | 4.11, p=0.001 | 2.66, p=0.068 |
| Light - Square | 8.40, p<0.001 | 7.88, p<0.001 | 5.59, p<0.001 | 3.32, p=0.011 |
| Light - Stripe | 1.82, p=0.366 | 12.01, p<0.001 | 3.21, p=0.015 | 3.04, p=0.025 |
| Square - Stripe | 6.57, p<0.001 | 4.13, p=0.001 | 2.39, p=0.128 | 0.28, p=0.999 |

(b) Proportion of trials with errors (values are t statistics with df = 96 and associated p-values).

| Experiment: | Greyscale | Red-Green | Yellow-Blue | |
| --- | --- | --- | --- | --- |
| Square size: | Both | Both | Small | Large |
| **Border - Dark**^1^ | **4.51, p<0.001** | **3.97, p<0.001** | **1.26, p=0.210** | **Not computable** |
| **Border - Light**^1^ | **3.87, p<0.001** | **3.43, p=0.001** | **1.72, p=0.090** | **3.01, p=0.003** |
| **Border - Square**^1^ | **3.48, p=0.001** | **2.91, p=0.004** | **1.52, p=0.131** | **3.25, p=0.001** |
| **Border - Stripe**^1^ | **4.38, p<0.001** | **2.50, p=0.014** | **1.41, p=0.162** | **3.07, p=0.002** |
| Dark - Light | 2.19, p=0.170 | 0.91, p=0.893 | 0.56, p=0.980 | Not computable |
| Dark - Square | 1.46, p=0.563 | 6.50, p<0.001 | 0.33, p=0.997 | Not computable |
| Dark - Stripe | 0.11, p=1.000 | 4.11, p=0.001 | 0.22, p=0.999 | Not computable |
| Light - Square | 2.81, p=0.040 | 5.59, p<0.001 | 0.24, p=0.999 | 0.74, p=0.934 |
| Light - Stripe | 2.24, p=0.155 | 3.21, p=0.015 | 0.33, p=0.997 | 0.19, p=0.999 |
| Square - Stripe | 1.34, p=0.641 | 2.39, p=0.128 | 0.10, p=1.000 | 0.55, p=0.977 |

^1^Comparisons with Border (in bold) are simple contrasts of *a priori* interest and so without control for multiple testing. All other tests are secondary and have Tukey-type control for multiple testing.
